# Implementation of a Randomized, Placebo-Controlled Trial of Live Attenuated Malaria Sporozoite Vaccines in an Indonesian Military Study Population

**DOI:** 10.4269/ajtmh.23-0597

**Published:** 2024-03-26

**Authors:** Khoriah Indrihutami, Krisin Chand, Rizka Fahmia, Mutia Rahardjani, Fitria Wulandari, Decy Subekti, Rintis Noviyanti, Amin Soebandrio, Noch T. Mallisa, I Made Mardika, Waras Budiman, Irwan Suriswan, Yogi Ertanto, Mei-Chun Chen, Tooba Murshedkar, Yonas Abebe, B. Kim Lee Sim, Stephen L. Hoffman, Thomas L. Richie, Sky Chen, Iqbal R. F. Elyazar, Lenny L. Ekawati, J. Kevin Baird, Erni J. Nelwan

**Affiliations:** ^1^ Oxford University Clinical Research Unit Indonesia, Jakarta, Indonesia;; ^2^ Eijkman Research Center for Molecular Biology, National Research & Innovation Agency, Cibinong, West Java, Indonesia;; ^3^ EXEINS Health Initiative, Jakarta, Indonesia;; ^4^ Faculty of Medicine, Universitas Indonesia, Jakarta, Indonesia;; ^5^ Presidential Staff Office, Republic of Indonesia, Jakarta, Indonesia;; ^6^ Gatot Soebroto Army Hospital, Jakarta, Indonesia;; ^7^ Muhammadiyah University, Surabaya, East Java, Indonesia;; ^8^ Army Medical Center, Army of the Republic of Indonesia, Jakarta, Indonesia;; ^9^ Sanaria Inc., Rockville, Maryland;; ^10^ StatPlus, Inc. Taipei, Taiwan;; ^11^ Centre for Tropical Medicine & Global Health, Nuffield Department of Medicine, University of Oxford, Oxford, United Kingdom;; ^12^ Division of Tropical Medicine and Infectious Disease, Department of Internal Medicine, Cipto Mangunkusumo Hospital, Jakarta, Indonesia

## Abstract

Malaria eradication efforts prioritize safe and efficient vaccination strategies, although none with high-level efficacy against malaria infection are yet available. Among several vaccine candidates, Sanaria^®^ PfSPZ Vaccine and Sanaria PfSPZ-CVac are, respectively, live radiation- and chemo-attenuated sporozoite vaccines designed to prevent infection with *Plasmodium falciparum*, the leading cause of malaria-related morbidity and mortality. We are conducting a randomized normal saline placebo-controlled trial called IDSPZV1 that will analyze the safety, tolerability, immunogenicity, and efficacy of PfSPZ Vaccine and PfSPZ-CVac administered pre-deployment to malaria-naive Indonesian soldiers assigned to temporary duties in a high malaria transmission area. We describe the manifold challenges of enrolling and immunizing 345 soldier participants at their home base in western Indonesia before their nearly 6,000-km voyage to eastern Indonesia, where they are being monitored for incident *P. falciparum* and *Plasmodium vivax* malaria cases during 9 months of exposure. The unique regulatory, ethical, and operational complexities of this trial demonstrate the importance of thorough planning, frequent communication, and close follow-up with stakeholders. Effective engagement with the military community and the ability to adapt to unanticipated events have proven key to the success of this trial.

## INTRODUCTION

Malaria kills more than 600,000 people annually.^1^ *Plasmodium*-bearing anopheline mosquitoes carry the infection. *Plasmodium falciparum* (Pf), *P. vivax* (Pv), *P. malariae*, and *P. ovale* infect humans as their intermediate host.^2^^,^^3^ Among these many plasmodia infecting humans, Pf and Pv dominate the global burden of morbidity, and Pf is the leading cause of mortality.^1^

The WHO’s Global Technical Strategy for malaria control and elimination aims to reduce malaria mortality and case incidence by 90% by 2030 compared with 2015.^4^ Malaria vaccines will play an important role in enabling and realizing this ambitious goal. The WHO has recommended using the vaccine RTS,S/AS01 (Mosquirix^TM^ GSK^®^, Brentford, UK) in infants and children to prevent serious illness caused by Pf; however, the expressed need for malaria vaccines with ≥90% protective efficacy against infection has yet to be achieved, and research to provide this level of efficacy is ongoing. The WHO listed 89 malaria vaccine candidates and 77 clinical trials in July 2022; 52 are completed, and 25 are ongoing, five of which involve Sanaria^®^ (Rockville, MD) PfSPZ Vaccine or PfSPZ-CVac.^5^

Unlike RTS,S/ASO1 and most other vaccine candidates, PfSPZ Vaccine and PfSPZ-CVac use live, infectious sporozoites (SPZ) of Pf as the immunogen. PfSPZ Vaccine is comprised of radiation-attenuated PfSPZ and PfSPZ-CVac of fully viable and infectious PfSPZ attenuated in vivo by chemoprophylaxis during immunization, which in the case of the Indonesian soldiers’ trial is the blood schizonticidal drug chloroquine (CQ).^6^ The PfSPZ for both vaccines are produced under the Current Good Manufacturing Practice, harvested from aseptic anopheline mosquitoes, and stored in liquid nitrogen vapor phase.^7^ Richie et al. explained the scientific progress made with whole sporozoite vaccination.^8^ The most recent PfSPZ Vaccine trial findings in Burkina Faso revealed 48% and 46% vaccine efficacy in adults against naturally transmitted Pf infection at 6 and 18 months, respectively, and the most recent PfSPZ-CVac (CQ) trial findings showed 100% vaccine efficacy against heterologous controlled human malaria infection at 12 weeks after immunization in malaria-naive adults.^9^^,^^10^

As an effort to provide evidence in guiding the rational selection and use of vaccine products for malaria hypo- to meso-endemic nations like Indonesia, Sanaria, Inc. and the University of Oxford (United Kingdom) signed a clinical trial agreement in 2017 to conduct a phase 2 trial, called IDSPZV1, of both PfSPZ Vaccine and PfSPZ-CVac products. This article describes the IDSPZV1 study design, as well as the challenges associated with obtaining ethics and regulatory approvals and investigational product importation permits and with choosing and engaging an infantry battalion and conducting study initiation, recruitment, and, finally, immunization. The sensitive task of enrolling and managing trial participants in a military setting demanded special implementation strategies.

### Study management and organization.

#### Start-up and stakeholder engagement.

Preparations for the IDSPZV1 trial began in 2015 with discussions between the leadership of Sanaria Inc. and Oxford University Clinical Research Unit (OUCRU) Indonesia, which led to a joint funding proposal in 2016 that was unsuccessful despite strong support from three institutions: the Eijkman Institute for Molecular Biology (EIMB; now the Eijkman Research Center for Molecular Biology under the National Research and Innovation Agency/Badan Riset dan Inovasi Nasional), the Commander of Health Services Headquarters of the Indonesian Army, and the Faculty of Medicine Universitas Indonesia. The funding deficit was overcome when Sanaria Inc. received support from the Peer Review Medical Research Program under the Congressionally Directed Medical Research Program in early 2017 to conduct IDSPZV1 with the same partners. Later that year, Sanaria Inc. and the Center for Tropical Medicine and Global Health, Nuffield Department of Medicine, University of Oxford signed a clinical trial agreement. The former EIMB and the Indonesian Army subsequently signed a cooperative agreement in early 2017. Due to reorganizations within the Indonesian Army Health Services Headquarters and disruptions due to the COVID-19 pandemic, a letter of agreement was not signed until June 2021. In November 2021, the study team evaluated scheduled battalion deployments and identified a battalion based at Bangkinang, Riau, Sumatra, that was scheduled for duty in the Keerom District of northeastern Papua in September 2022. This selection was guided by none-to-negligible malaria transmission at home base, a high-transmission temporary duty station, and goodness of calendar-fit (soldiers available >5 months before deployment).

#### Ethical and regulatory oversight.

The Faculty of Medicine Universitas Indonesia Ethics Committee in Jakarta (0228/UN2.F1/ETIK/2018), the Oxford Tropical Research Ethics Committee in the United Kingdom (OXTREC 13-18), and National Agency of Food and Drug Control Republik Indonesia (Badan Pengawas Obat dan Makanan [BPOM RI] in Jakarta (B-PN.1.06.3.321.05.19.1739) approved the trial. The trial was conducted under an Investigational New Drug Application allowed by the U.S. Food and Drug Administration (FDA). All clearances were granted in 2018 and 2019, respectively. However, due to COVID-19 and other delays, the study team resubmitted all applications in 2021, including a new application to the Gatot Soebroto Army Hospital Medical Committee in Jakarta, for renewals and relevant protocol amendments. In March 2022, the BPOM issued the second approval and import licenses for the investigational product (IP). The trial is registered in ClinicalTrial.gov (identifier: NCT03503058).

#### Clinical trial team.

The principal (E. J. N., Faculty of Medicine, Universitas Indonesia) and responsible (J. K. B., OUCRU Indonesia) investigators oversaw 111 staff members at Bangkinang, Riau. Most OUCRU staff had clinical trial experience, and all renewed their Good Clinical Practice (GCP) certification and received training in the IDSPZV1 research protocol and trial standard operating procedures (SOPs). The sponsor provided a 2-week malaria microscopy course based on Sanaria’s U.S. FDA SOP for malaria diagnosis using thick blood film preparation and two independent reads. The Pharmaceutical Operations staff was trained in vaccine shipping, storage, thawing, and syringe preparation by Sanaria, with three staff members traveling to Rockville, MD, for initial instruction. Study clinicians received training from Sanaria staff traveling to Jakarta regarding direct venous inoculation (DVI), the preferred route of immunization for PfSPZ vaccines. Sponsor-led site qualification and site initiation visits conducted by an independent monitor occurred before the initiation of enrolment in early May 2022.

Except for one army doctor and six nurses, 42 military-assigned health workers in the battalion had no previous experience in research. Supplementary trainings were therefore provided by the OUCRU clinical and laboratory teams including clinical trials foundations, GCP, human research protection, good documentation practices, and case report form procedures. Clinical and laboratory procedures were also taught, including vital sign measurement & interpretation; basic malaria biology & chemotherapeutics; venipuncture and direct venous inoculation (DVI); blood smear preparation; and malaria rapid diagnostic test procedures.

#### Study socialization.

In March 2022, engagement with the battalion commanders began. Formal approval of the access to the battalion required ensuring subjects’ health and safety and accepting that clinical trial team members must reside temporarily within the army base and, later, within the area of operational deployment as embedded health professionals complementing the Army’s medical team. The commanders had to be assured that the trial was safe, had proper oversight and authorization, and would not interfere with the regular duties of the soldiers. The team, in turn, accepted the necessity that all members in the field must be Indonesian nationals.

#### Site logistics.

The calendar dominated all logistical considerations due to the projected date for the battalion’s departure to Papua. The research team identified mid-May 2022 as the latest possible commencement of vaccination to ensure immunization completion before soldiers’ embarkation. After obtaining all permits by early April 2022, the team started preparing the site at Sumatra. First, a clinical trial operations center was established at the army base in Bangkinang. The center included the following facilities: a functional medical clinic, a pharmacy operations laboratory and pharmacy dispensary, a fully outfitted clinical laboratory, a trial administration office, a logistical operations center, and living quarters for the team of 39 staff members from Jakarta. The team renovated existing buildings for these centrally located facilities. These were ready for occupation and use by late April 2022.

The IP, normal saline (NS) placebo, vaccine diluent, and associated supplies and equipment came to Jakarta from the United States and were forwarded to Bangkinang using World Courier, a specialized carrier for temperature-controlled logistics shipments. The cold chain for the IP includes all steps from setting up the dry shipper for shipment to the vaccine’s return from the clinical trial site after vaccination completion. The temperature is maintained at –150°C throughout both shipment and storage at the study site and is closely monitored with a temperature logger. Constant replenishment of liquid nitrogen was done weekly or as needed.

#### Trial commencement.

In early May 2022, all necessary items were ready for vaccination with the exception of CQ tablets for use with PfSPZ-CVac; the day before shipment, the UK supplier found the shipping agent was ineligible for pharmaceutical import to Indonesia. Therefore, the shipment was redirected to the University of Oxford where a team member flew to obtain it and returned to Indonesia with the CQ tablets as checked baggage. Through proper coordination with customs authorities and permits from the BPOM RI, the drugs were delivered to the site in Sumatra within 12 hours of arrival in Jakarta, allowing screening and enrollment to commence. The importance of these hours at the front end may be realized by examining the hours at the back end of this immunization phase of trial: the last post-vaccination follow-up visit for the last soldiers vaccinated occurred dockside immediately before embarkation to Papua.

### Study conduct.

#### Study design, setting, and population.

**Figure 1.**
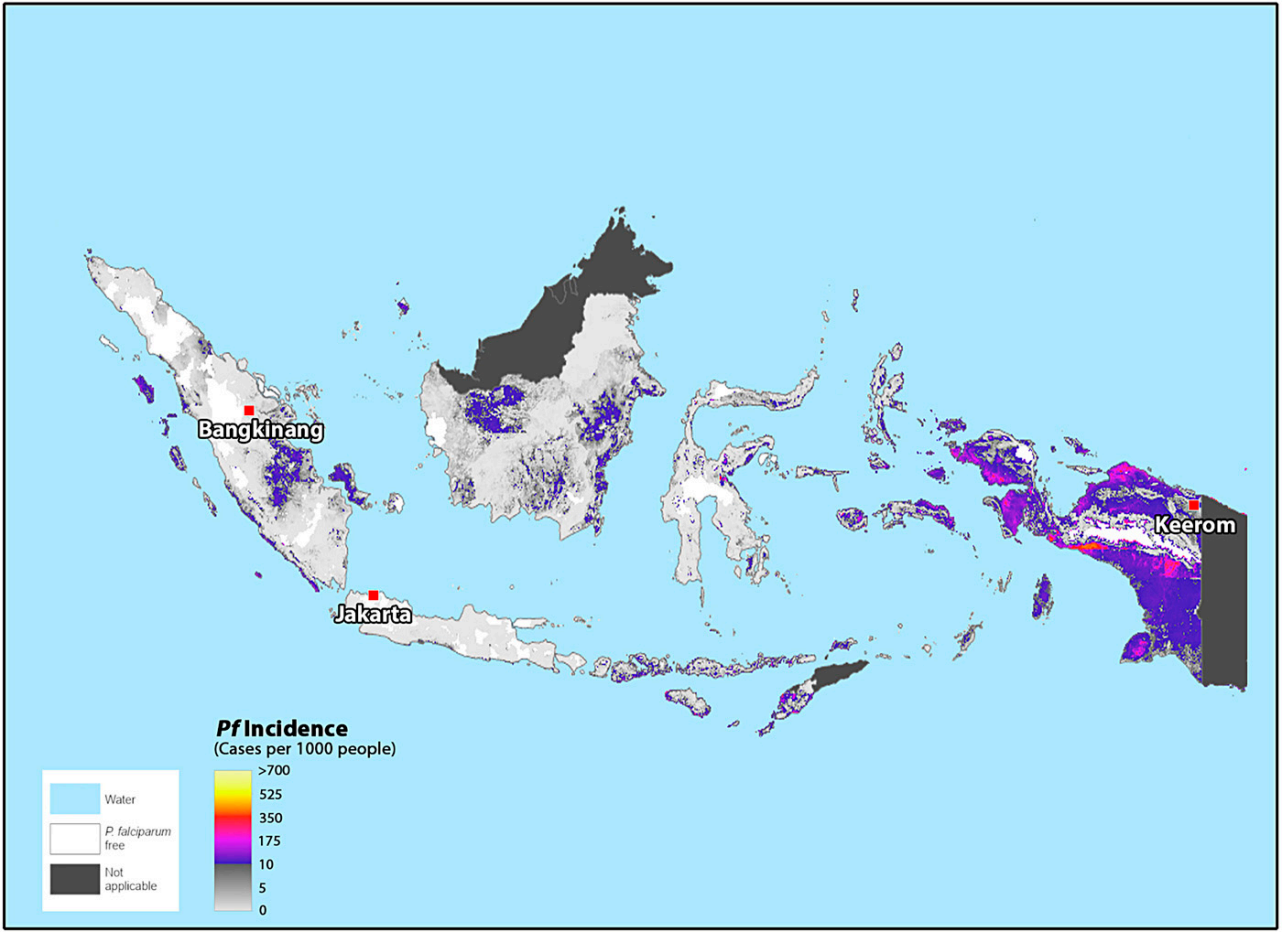
IDSPZV1 clinical trial sites in Indonesia and *Plasmodium falciparum* spatial distribution in 2020. Source: The Malaria Atlas Project, https://malariaatlas.org/.^11^

This double-blind, randomized, normal saline placebo-controlled, phase 2 clinical trial assesses the safety, tolerability, immunogenicity, and protective efficacy of PfSPZ Vaccine and PfSPZ-CVac against naturally occurring Pf and Pv malaria in healthy Indonesian soldiers deployed to eastern Indonesia. The study identified an infantry battalion based at Bangkinang in Sumatra, Indonesia, scheduled for a rotation of duty in a highly malarious region of eastern Indonesia, the Keerom area of northeastern Papua on the island of New Guinea (Figure 1).

Bangkinang city (37,247 inhabitants) in Riau province is 93.77 km^2^ with an average density of 789 residents/km^2^.^12^ Malaria cases among the 6.8 million residents of Riau were 0.12 per 1,000 people in 2021, with local transmission accounting for most cases. During the same year, Bangkinang reported 13 malaria cases, but just 5 were confirmed with laboratory diagnosis.^13^

#### Informed consent and eligibility.

The existence of military hierarchy intrinsically made our study population vulnerable to coercion and undue influence. To mitigate this risk, specific countermeasures were implemented before and during informed consent. During research socialization engagement, we emphasized the critical importance of voluntary participation and requested that the battalion commanders prevent soldiers from being ordered to participate or from being ostracized should they decline participation. The solicitation of informed consent by a trial physician was held in a private room and witnessed only by the prospective subject and an invited witness of equal or lower military rank where the same guiding principles of volunteerism were explained.

#### Screening and recruitment.

Over 5 days, 380 potential participants were screened. The chief medical officer held information sessions in groups of 10 to 12 soldiers before they were invited to a private room to express any additional concerns or questions before giving consent. One of the 380 invited soldiers declined consent, and 31 others proved ineligible, leaving 348 for randomization.

Once consented, study physicians performed clinical screening procedures. If a participant did not fulfill any nonlaboratory exclusion criteria, they advanced to venous blood sampling. Participants’ eligibility status was reviewed and confirmed after all laboratory results were formally reported, usually within 3–5 days after screening. Complete eligibility criteria are summarized in Table 1.

**Table 1:** Inclusion and exclusion criteria for IDSPZV1

Inclusion Criteria	Exclusion Criteria
Male aged 18–55 at screening	Previous vaccination with an investigational malaria vaccine
Assigned to the research battalion and deployed to eastern Indonesia	Use of an investigational or nonregistered drug or vaccine other than the study vaccine(s) within 30 days before the first study vaccination or planned use of up to 30 days after vaccination
Freely consents in writing to participate in the study	Chronic administration of immunosuppressant or other immune-modifying drugs within 6 months before the first vaccination
Agrees to follow Indonesian military medical guidance for malaria screening and treatment	Administration or planned administration of one live or three or more other type vaccinations 28 days before the first study vaccination and 28 days after the final vaccination
Physical examination and laboratory data without clinically significant findings and a body mass index ≤35 kg/m^2^	Immunosuppressive or immunodeficient state
	Autoimmune disease
	Chloroquine or other 4-aminoquinolone allergy or anaphylaxis
	Drug-related anaphylaxis or hospitalization
	Phosphate-buffered saline or human serum albumin allergy
	Use or planned use of any antimalarial drugs during the study except for antimalarial medication administered by study clinicians
	Splenectomy history
	Laboratory evidence of liver disease (the PI and clinical officers decide, but participant is excluded if any of the screening liver function tests (alanine transaminase, bilirubin, gamma gamma-glutamyl transferase) are more than double the upper limit of normal measured twice without an explanation for the abnormal values)
	Renal disease (serum creatinine >1.5 mg/dL, measured twice)
	Laboratory evidence of hematologic disease (platelet count or hemoglobin <80% of Indonesia’s lower limit, measured twice)
	Abnormal screening electrocardiogram showing prolonged QTc interval (>450 milliseconds), arrythmia/irregularity, ischemia, or cardiac enlargement considered suggestive of acute or chronic cardiovascular disease
	Acute or chronic pulmonary, cardiovascular, hepatic, renal or neurological condition, severe malnutrition, or other clinical findings assessed by the PI or her designee that may increase participating risk
	Immunoglobulin and/or any blood product administration within 3 months before the first study vaccination or planned administration during the study period
	Simultaneous participation in any other interventional clinical trial
	Other conditions that the PI or her designee believes may jeopardize participant’s safety, prevent them from following protocol, or affect data integrity
	Any active malaria, symptomatic or asymptomatic, confirmed by RDT, microscopy or real-time PCR before first injection of PfSPZ Vaccine or PfSPZ-CVac, unless treated by the clinical team
	Atypical or nonfebrile seizures
	Tuberculosis therapy
	Laboratory evidence of active infection with hepatitis B or C
	Participants with >10% 5-year cardiovascular risk (fatal and nonfatal) based on the Gaziano scoring system; participants in the 18–34-year age group were assessed as though they were in the 35–44-year age group
	Psychiatric disorders (e.g., personality disorders, anxiety disorders, or schizophrenia) or behavioral issues (including active alcohol or drug abuse) discovered during the screening

PCR = polymerase chain reaction; PI = principal investigator; RDT = rapid diagnostic test.

#### Randomization.

**Figure 2.**
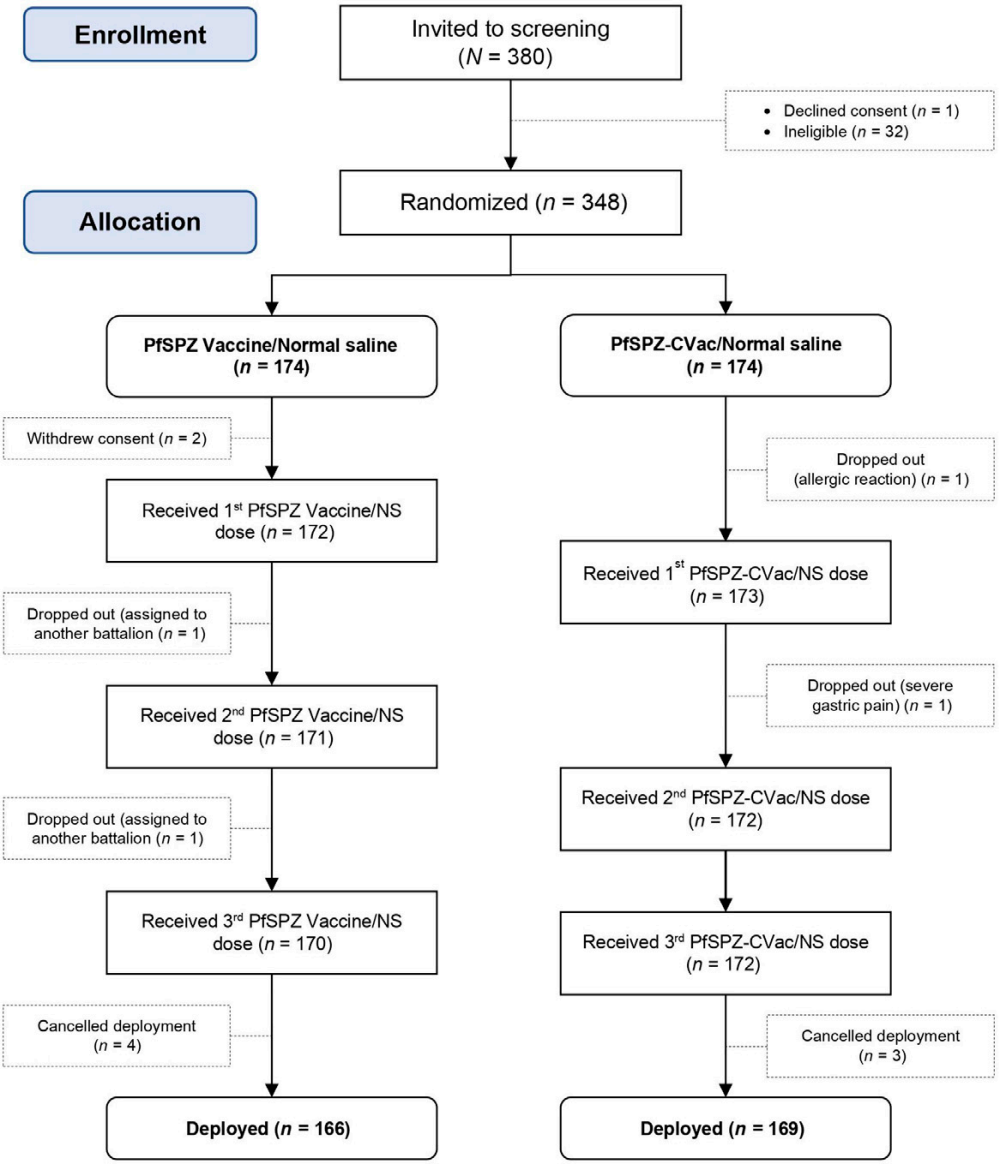
IDSPZV1 recruitment flow diagram.

The team enrolled and randomized 348 participants, of which 2 withdrew consent and 1 was dropped out before first immunization (Figure 2). First tier randomization consisted of the open label allocation of soldiers to either the PfSPZ Vaccine/NS group or the PfSPZ-CVac/NS group. This allocation could not be blinded because the vaccines are administered on different schedules, and CQ was administered only to the second group. On the first immunization day for each group, the unblinded syringe preparation staff (pharmaceutical operations team) performed the second-tier, blinded randomization. A variable block randomization algorithm developed by an independent statistician generated a master randomization list to assign members to vaccine or NS, based on a 2:1 ratio. Group 1 received PfSPZ Vaccine on days 1, 8, and 29; Group 2, NS on days 1, 8, and 29 as a control for Group 1; Group 3, PfSPZ-CVac on days 1, 29, and 57 with weekly CQ prophylaxis starting on day –2 before the first vaccination and extending to the week after the last vaccination; and Group 4, NS on days 1, 29, and 57, also with CQ, as a control for Group 3.

#### Vaccination and CQ dosing.

PfSPZ Vaccine and the immunogen for PfSPZ-CVac, called PfSPZ Challenge are manufactured by Sanaria, Inc. at their GMP facilities in Rockville, MD. PfSPZ Vaccine consists of radiation-attenuated PfSPZ, whereas PfSPZ-CVac (CQ) consists of viable *P. falciparum* sporozoites (PfSPZ Challenge™). Three doses of 9 × 10^5^ PfSPZ of PfSPZ Vaccine and three doses of 2 × 10^5^ PfSPZ of PfSPZ Challenge were administered through DVI to participants assigned to Groups 1 and 3. The PfSPZ of PfSPZ Vaccine are unable to replicate in the liver, whereas the PfSPZ of PfSPZ Challenge replicate normally, expanding the biomass and diversity of the immunogen, inducing equal or greater protective immunity using a smaller dose. The PfSPZ-CVac/NS group took Avloclor (Alliance Pharmaceuticals Ltd., Chippenham, United Kingdom) tablets containing 250 mg of CQ phosphate (150 mg of CQ base). Two days before the first PfSPZ-CVac/NS dose, CQ base was loaded at 10 mg/kg. Thereafter, CQ was administered orally in 9 weekly doses of 5 mg/kg under direct observation until 5 days after the last dose.

### Vaccination period follow-up.

#### Adverse events follow-up.

##### PfSPZ Vaccine/NS group.

Participants were invited to the study clinic on day +2 for local and systemic adverse event (AE) assessment and +7 for systemic AE assessment. Physicians examined for tenderness, induration, bruising/extravasated blood, erythema, and swelling and asked about pain and pruritus at the injection site, as the solicited local AEs. Participants were asked about subjective fever, signs or symptoms consistent with allergic reaction (rash, urticaria, pruritus, edema), headache, subjective fever, fatigue, malaise, chills, myalgia, and arthralgia as the solicited systemic AEs. Body temperature was also monitored, with temperatures >37.5°C considered to be fever. Unsolicited systemic AEs were also observed until day +14 after each dose.

##### PfSPZ-CVac/NS group.

PfSPZ-CVac/NS participants had the same local solicited AEs noted for +2 days after each dose. Solicited systemic AEs were followed from day –2 to day 69 (+7 days after final CQ dose) and for unsolicited AEs from day –2 to day 71 (+14 days after last PfSPZ-CVac/NS dose). This more intensive evaluation was based on the transient parasitemia that occurs in PfSPZ-CVac (CQ) recipients on days +7, +8, and +9, particularly after the first dose and ongoing use of CQ. To monitor AEs potentially associated with transient parasitemia and CQ, more systemic AEs were solicited in addition to the core list: dizziness, rigors, sweats, cough, nausea, vomiting, abdominal pain, diarrhea, chest pain, palpitations, shortness of breath, tinnitus, blurred vision, photosensitivity, insomnia, pruritus, anxiety, and confusion.

#### Laboratory follow-up.

Follow-up visits included clinical examination and blood collection for laboratory analysis. Tables 2 and 3 show PfSPZ Vaccine and PfSPZ-CVac study procedures during vaccination.

**Table 2:** Procedures schedule for the PfSPZ Vaccine/normal saline group

PfSPZ Vaccine Group	Screening Period	Vaccination Period (pre-exposure)						
		43 days						
Study weeks relative to first vaccination	−1	1	2	3	4	5	6	7
Study days relative to first vaccination	−2	1	8	15	22	29	36	43
Study visit	SC	V1	V2 (V1 + 7)	V2 + 7	V2 + 14	V3	V3 + 7	V3 + 14
Visit windows	−56	–	±1	±1	±2	±4	±1	±2
PfSPZ Vaccine administration		DVI1	DVI2			DVI3		
Clinical assessment								
Informed consent	x							
Eligibility review	X	x	x			x		
Medical history	x							
Assignment of study ID	x							
Enrollment and randomization	x^4^							
Physical examination								
Detailed	x							
Focused*		x	x	x	x	x	x	x
Physical assessment								
Vital signs	x	2x^†^	2x^†^	x	x	2x^†^	x	x
Weight and height	x							
Electrocardiogram	x							
AE data collection								
Solicited		x	x	x		x	x	
Unsolicited		x	x	x	x	x	x	x
Serious AE and other		x	x	x	x	x	x	x
Medication history/concomitant medication	x	x	x	x	x	x	x	x
Laboratory assessment								
Hematology and biochemistry^‡^	x^§^	x						x
Parasitology								
Thick and thin blood smear	x	x						x
Qualitative PCR	x							x
Parasite genome seq: WGA filter paper	x	x						x
Parasite genome seq: venous sample		x						x
Urinalysis	x							
G6PD and serology (HBV, HCV)	x							
Immunology								
Humoral		x						x
Cellular		x			x			x
Troponin		x						

AE = adverse event; DVI = direct venous inoculation; HBV = hepatitis B virus; HCV = hepatitis C virus; PCR = polymerase chain reaction; WGA = wheat germ agglutinin.

After Screening Visit 1 and screening results review, eligible volunteers were enrolled and randomized to one of four treatment group.

* Focused on brief medical condition review.

^†^ On vaccination days, vital signs were taken twice: before vaccination to ensure eligibility and at least 30 minutes post-vaccination.

^‡^ Hematology/biochemistry included complete blood count, creatinine, alanine transaminase (glutamic oxaloacetic transaminase).

^§^ Biochemistry included bilirubin, gamma-glutamyl transferase, and glucose only during screening.

**Table 3:** Procedures schedule for the PfSPZ-CVac/NS group

PfSPZ-CVac Group	Screening Period		Vaccination Period (pre-exposure)																					
			71 Days																					
Study week relative to first vaccination	−1	−1	1		2				3	4	5		6				7	8	9		10			11
Study day relative to first vaccination	−4	−2	1	6	8	9	10	13	20	27	29	34	36	37	38	41	48	55	57	62	64	65	66	71
Study visit	SC	CV-2	CV1	CV1 + 5	CV1 + 7	CV1 + 8	CV1 + 9	CV1 + 12	CV1 + 19	CV1 + 26	CV2	CV2 + 5	CV2 + 7	CV2 + 8	CV2 + 9	CV2 + 12	CV2 + 19	CV2 + 26	CV3	CV3 + 5	CV3 + 7	CV3 + 8	CV3 + 9	CV3 + 14
Visit windows	−56	±1	-	±2	-	-	-	±2	±2	±2	±7*	±2	–	–	–	±2	±2	±2	±7*	±2	–	–	–	±3
PfSPZ-CVac administration			DVI1								DVI2								DVI3					
Antipyretic drug administration^†^					x	x							x	x							x	x		
CQ dosing, mg		620		310				310	310	310		310				310	310	310		310				
Clinical assessment																								
Informed consent	X																							
Eligibility review	x	x	x								x								x					
Medical history	x																							
Assignment of study ID	x																							
Enrollment and randomization^‡^	x																							
Physical examination																								
Detailed	x																							
Focused^§^		x	x	x	x	x	x	x	x	x	x	x	x	x	x	x	x	x	x	x	x	x	x	x
Physical assessment																								
Vital signs	x	x	2x^‖^	x	x	x	x	x	x	x	2x^‖^	x	x	x	x	x	x	x	2x^‖^	x	x	x	x	x
Weight and height	x																							
Electrocardiogram	x																							
AE data collection																								
Solicited^¶^		x		x	x	x	x	x	x	x	x	x	x	x	x	x	x	x	x	x	x	x	x	x^4^
Unsolicited		x		x	x	x	x	x	x	x	x	x	x	x	x	x	x	x	x	x	x	x	x	x
Serious AE and others		x	x	x	x	x	x	x	x	x	x	x	x	x	x	x	x	x	x	x	x	x	x	x
Medication history/concomitant medication	x	x	x	x	x	x	x	x	x	x	x	x	x	x	x	x	x	x	x	x	x	x	x	x
Laboratory assessment																								
Hematology and biochemistry^#^^,^**	x		x																					x
Parasitology																								
Thick and thin blood smear	x		x		x	x	x	x			x		x	x	x	x			x		x	x	x	x
qPCR	X		x		x	x	x	x			x		x	x	x	x			x		x	x	x	x
Parasite genome seq: WGA filter paper	x		x		x	x	x	x			x		x	x	x	x			x		x	x	x	x
Parasite genome seq: venous sample			x																					x
Urinalysis	x																							
G6PD and serology (HBV, HCV)	x																							
Immunity																								
Humoral			x																					x
Cellular			x					x																x
Troponin			x																					

AE = adverse event; DOT = directly observed treatment; DVI = direct venous inoculation; HBV = hepatitis B virus; HCV = hepatitis C virus; PCR = polymerase chain reaction; WGA = wheat germ agglutinin.

* Add, decrease, or alter chloroquine (CQ) dose if moved > 2 days.

^†^ DOT administered the morning doses each day, and study subjects took doses later in the day on their own.

^‡^ If eligible, volunteers were enrolled and randomly assigned to one of four treatment groups after Screening Visit 1.

^§^ Brief medical review.

^‖^ Before and after vaccination, vital signs were taken to establish eligibility.

^¶^ Solicited AE collection was +7 days post-CQ administration on Day 69, but it would be done on Day 71.

^#^ Hematology/biochemistry included complete blood count, creatinine, alanine transaminase (glutamic oxaloacetic transaminase).

** Bilirubin, gamma glutamyl transferase, and glucose were only tested at screening.

From May 7 to October 1, 2022, soldiers were engaged, enrolled, and vaccinated before departing for Papua. None were positive for malaria by thick blood smear or polymerase chain reaction. The research team has now followed the battalion to Papua to surveil the soldiers continuously for naturally acquired Pf and Pv malaria infections through 9 months of exposure (not described here).

### Challenges and solutions.

Implementing a complex trial in a dynamic military setting provided unique challenges as well as opportunities to the team. Here we highlight a few challenges encountered during study conduct and the strategies we implemented to address them.

**Figure 3.**
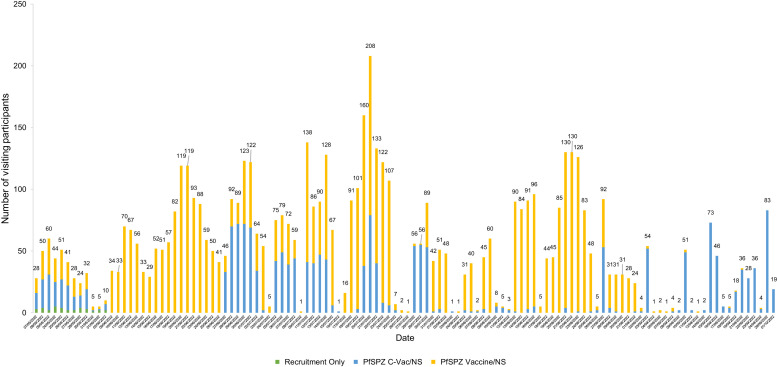
IDSPZV1 study team workload.

Figure 3 provides a visual representation of the team workload at Bangkinang, which consisted of daily, tight and overlapping procedures. To distribute the workload, participants in each main group were divided into five cohorts, resulting in varied numbers of visiting participants and workload each day.

#### Revised vaccination timeline.

The PfSPZ Vaccine/NS and PfSPZ-CVac/NS groups were supposed to be vaccinated within 7 and 11 weeks, respectively. In May 2022, knowing soldiers would be deployed in July, screening began. Fortunately, the deployment date was postponed multiple times, as we encountered issues that necessitated adjustment to the vaccination timelines, and the delayed departure dates fortuitously allowed these adjustments.

For PfSPZ-CVac/NS, the first CQ and PfSPZ-CVac/NS dose were administered during the 2nd week of June 2022 over the course of 5 days. The second PfSPZ-CVac/NS dose was given on time, but the third dose was given 5 days late due to a brief immunization hold (described subsequently). This necessitated the administration of an additional CQ dose (11 and 12 doses in total rather than the planned 10 doses, for some participants).

For PfSPZ Vaccine/NS, the first dose of PfSPZ Vaccine/NS began 3 weeks later than the PfSPZ-CVac/placebo. This timing was intended to complete immunizations in both groups at roughly the same time. The second PfSPZ Vaccine/NS dose was administered on time, but further immunizations were then halted by the sponsor for technical reasons. After vaccination notification resumed, administration of the third dose was partially completed 4 weeks later. This was halted by a second sponsor request. After 3 weeks, immunization resumed, and 118 participants received their final PfSPZ vaccine dose a week before deployment (a delay of 7 weeks). Because of time constraints, the post-vaccination laboratory samples for these participants were taken out of window, and clinical follow-ups were done by phone calls during the voyage to Papua.

Of note, both study halts involved the primary ethics committees, BPOM, and the U.S. FDA, with all institutions concurring each time that vaccinations could safely resume.

#### Off-site visits.

Engaging military subjects with dynamic duty schedules required flexibility and dedication in planning participant schedules to minimize out-of-window and missed visits. The study team had to be fully prepared for unanticipated off-site visits such as when all soldiers were assigned to 18 isolated locations for pre-deployment training for 2 weeks. During this period, all clinical and laboratory procedures were done on the training ground.

#### Clinical care.

The clinical team established a 24-hour on-call system where health workers could notify the study physicians of any occurrence of AEs. Decisions for referrals of severe or serious cases were made collaboratively with the army health commanders. The team and army health platoon identified Bangkinang Regional Public Hospital, 8 km from the base, to be referrals of emergency and other clinical cases. However, more complex cases often required referrals to larger healthcare facilities, including the Army Hospital in Pekanbaru, which required up to 2 hours of extra travel time.

#### Logistics and procurements.

The team had to prepare for the constant replenishment of supplies and people throughout the 5 months at Bangkinang without allowance for a pause of any sort. This required daily interactions with OUCRU headquarters in Jakarta and frequent shipments to Sumatra to maintain critical inventories.

#### Deployment preparation.

The soldiers embarked to Papua on a 28-day ship journey, without direct contact with the study team due to logistical and safety concerns. The army health workers were therefore the most reliable source of assistance for clinical management and data recording. To ensure participants’ safety and accuracy of data recording during voyage, the study team trained the army health workers in clinical examination, malaria diagnosis and management, and filling out translated case report forms. In addition, working flowcharts, medical and laboratory supplies, and anti-malaria medications were also provided.

## CONCLUSION

This international trial experience occurred in the face of daunting ethical, regulatory, and logistical complexities, which challenged both the research team and Sanaria, the trial sponsor. Its ultimate success relied on effective engagement, communications, and coordination across private, academic, and national research institutions, three governing ethics committees, two national regulatory authorities, along with the Indonesian Ministries of Health and Defense. That essential work laid the foundation on which the clinical research team and its many partners carried out the GCP vaccination of 345 soldiers with two distinct vaccine formulations and saline placebos immediately before their departure for 9 months of security duties in a highly malarious area of eastern Indonesia. This enterprise carried substantial risks of failure, including, loss of the trust and good will needed to access a military population, unchecked coercion in a military hierarchical setting, partial vaccination before departure, and emergency redirection of the infantry battalion to other duties.

The acceptance of these risks and the perseverance by all stakeholders allowed for the ultimate success of the now completed IDSPZV1 immunization phase. Those involved understood the importance of what IDSPZV1 promises to deliver—a host of firsts: 1) the first malaria vaccine trial in the Asia Pacific region in more than a quarter of a century; 2) the first examination of PfSPZ vaccine technologies against non-African *P. falciparum*; 3) the first look at PfSPZ vaccine technologies against *P. vivax* anywhere; 4) the first examination of PfSPZ vaccine technologies against naturally acquired *P. falciparum* in nonimmune adult subjects; and 5) the first head-to-head examination of radiation- versus drug-attenuated PfSPZ vaccine formulations for field protection, which is likely to inform development strategy directly.

The motivation of the Indonesian authorities not only to allow the trial but also to endure its risks and delays may be useful to understand. Malaria is a serious and ongoing threat to millions of Indonesians, especially members of its armed forces who work in high-risk settings. The army is keen to develop practical solutions to malaria threatening its personnel. Likewise, the Ministry of Health has declared a goal of malaria elimination in a stepwise fashion in the coming decades and sees vaccines preventing malaria infection as a potentially useful tool in realizing this aim. However, the global focus of vaccine research on the African malaria problem in infants and young children offers little directly relevant evidence for the malaria problem in people of all ages and relatively little naturally acquired immunity. Which vaccines may be most useful in the Indonesian setting, civilian and military personnel alike, is an open question that is currently lacking direct evidence. Ideally, the IDSPZV1 trial will begin to close that gap and inform real strategies with robust and relevant clinical evidence.
