# Authorship Correction: Implementation of a Randomized, Placebo-Controlled Trial of Live Attenuated Malaria Sporozoite Vaccines in an Indonesian Military Study Population

**DOI:** 10.4269/ajtmh.23-0597cor

**Published:** 2024-08-07

In the published original research paper, “Implementation of a Randomized, Placebo-Controlled Trial of Live Attenuated Malaria Sporozoite Vaccines in an Indonesian Military Study Population” by Indrihutami et al., 2024 (https://doi.org/10.4269/ajtmh.23-0597), the first author identified a clerical error of four coauthor omissions. The paper has therefore been corrected with the addition of the four COPE compliant and verified coauthors.

A summary of the corrected data is shown here:

1. Author byline was changed, adding Agus Rachmat, Inge Sutanto, Eric R. James, and Peter F. Billingsley as follows:
  “Khoriah Indrihutami,^1^ Krisin Chand,^1^ Rizka Fahmia,^1^ Mutia Rahardjani,^1^ Fitria Wulandari,^1^ Decy Subekti,^1^ Rintis Noviyanti,^2,3^ Amin Soebandrio,^4^ Noch T. Mallisa,^5^ I Made Mardika,^6^ Waras Budiman,^7^ Irwan Suriswan,^6^ Yogi Ertanto,^8^ Mei-Chun Chen,^9^ Tooba Murshedkar,^9^ Yonas Abebe,^9^ B. Kim Lee Sim,^9^ Stephen L. Hoffman,^9^ Thomas L. Richie,^9^ Sky Chen,^10^ Iqbal R.F. Elyazar,^1^ Lenny L. Ekawati,^1,11^ J. Kevin Baird,^1,11^ and Erni J. Nelwan^4,12^*”
to “Khoriah Indrihutami,^1^ Krisin Chand,^1^ Rizka Fahmia,^1^ Agus Rachmat,^1^ Mutia Rahardjani,^1^ Fitria Wulandari,^1^ Decy Subekti,^1^ Rintis Noviyanti,^2,3^ Inge Sutanto,^4^ Amin Soebandrio,^4^ Noch T. Mallisa,^5^ I Made Mardika,^6^ Waras Budiman,^7^ Irwan Suriswan,^6^ Yogi Ertanto,^8^ Mei-Chun Chen,^9^ Tooba Murshedkar,^9^ Yonas Abebe,^9^ Eric R. James,^9^ Peter F. Billingsley,^9^ B. Kim Lee Sim,^9^ Stephen L. Hoffman,^9^ Thomas L. Richie,^9^ Sky Chen,^10^ Iqbal R.F. Elyazar,^1^ Lenny L. Ekawati,^1,11^ J. Kevin Baird,^1,11^ and Erni J. Nelwan^4,12^*” (Title page)
2. Authors’ addresses section pertaining to current contact information was changed from “Authors’ addresses: Khoriah Indrihutami, Krisin Chand, Rizka Fahmia, Mutia Rahardjani, Fitria Wulandari, Decy Subekti, and Iqbal R.F. Elyazar, Oxford University Clinical Research Unit Indonesia, Jakarta, Indonesia, E-mails: kindrihutami@oucru.org, sanariliskris@yahoo.com, rfahmia@oucru.org, mrahardjani@oucru.org, fwulandari@oucru.org, dsubekti@oucru.org, and ielyazar@oucru.org. Rintis Noviyanti, Eijkman Research Center for Molecular Biology, National Research & Innovation Agency, Cibinong, West Java, Indonesia, and EXEINS Health Initiative, Jakarta, Indonesia, E-mail: r.noviyanti@exeins.org. Amin Soebandrio, Faculty of Medicine, Universitas Indonesia, Jakarta, Indonesia, E-mail: amin.subandrio@ui.ac.id. Noch T. Mallisa, Presidential Staff Office, Republic of Indonesia, Jakarta, Indonesia, E-mail: nochtiranduk1@gmail.com. I Made Mardika and Irwan Suriswan, Gatot Soebroto Army Hospital, Jakarta, Indonesia, E-mails: immardika@gmail.com and irwansuriswan@gmail.com. Waras Budiman, Muhammadiyah University, Surabaya, Indonesia, E-mail: waras.budiman4@gmail.com. Yogi Ertanto, Army Medical Center, Army of the Republic of Indonesia, Jakarta, Indonesia, E-mail: joghie614@gmail.com. Mei-Chun Chen, Tooba Murshedkar, Yonas Abebe, B. Kim Lee Sim, Stephen L. Hoffman, and Thomas L. Richie, Sanaria Inc., Rockville, MD, E-mails: mchen@sanaria.com, tmurshedkar@sanaria.com, yabebe@sanaria.com, ksim@sanaria.com, slhoffman@sanaria.com, and trichie@sanaria.com. Sky Chen, StatPlus, Inc. Taipei, Taiwan, E-mail: sky.chen@statplus.com. Lenny L. Ekawati and J. Kevin Baird, Oxford University Clinical Research Unit Indonesia, Jakarta, Indonesia, and Centre for Tropical Medicine & Global Health, Nuffield Department of Medicine, University of Oxford, Oxford, United Kingdom, E-mails: lekawati@oucru.org and kbaird@oucru.org. Erni J. Nelwan, Faculty of Medicine, Universitas Indonesia, Jakarta, Indonesia, and Division of Tropical Medicine and Infectious Disease, Department of Internal Medicine, Cipto Mangunkusumo Hospital, Jakarta, Indonesia, E-mail: ejnelwan@yahoo.com.”
to “Authors’ addresses: Khoriah Indrihutami, Krisin Chand, Rizka Fahmia, Agus Rachmat, Mutia Rahardjani, Fitria Wulandari, Decy Subekti, and Iqbal R.F. Elyazar, Oxford University Clinical Research Unit Indonesia, Jakarta, Indonesia, E-mails: kindrihutami@oucru.org, sanariliskris@yahoo.com, rfahmia@oucru.org, arachmat@oucru.org, mrahardjani@oucru.org, fwulandari@oucru.org, dsubekti@oucru.org, and ielyazar@oucru.org. Rintis Noviyanti, Eijkman Research Center for Molecular Biology, National Research & Innovation Agency, Cibinong, West Java, Indonesia, and EXEINS Health Initiative, Jakarta, Indonesia, E-mail: r.noviyanti@exeins.org. Amin Soebandrio and Inge Sutanto, Faculty of Medicine, Universitas Indonesia, Jakarta, Indonesia, E-mails: amin.subandrio@ui.ac.id, inge.sutanto@ui.ac.id. Noch T. Mallisa, Presidential Staff Office, Republic of Indonesia, Jakarta, Indonesia, E-mail: nochtiranduk1@gmail.com. I Made Mardika and Irwan Suriswan, Gatot Soebroto Army Hospital, Jakarta, Indonesia, E-mails: immardika@gmail.com and irwansuriswan@gmail.com. Waras Budiman, Muhammadiyah University, Surabaya, Indonesia, E-mail: waras.budiman4@gmail.com. Yogi Ertanto, Army Medical Center, Army of the Republic of Indonesia, Jakarta, Indonesia, E-mail: joghie614@gmail.com. Mei-Chun Chen, Tooba Murshedkar, Eric R. James, Peter F. Billingsley, Yonas Abebe, B. Kim Lee Sim, Stephen L. Hoffman, and Thomas L. Richie, Sanaria Inc., Rockville, MD, E-mails: mchen@sanaria.com, tmurshedkar@sanaria.com, ejames@sanaria.com, pbillingsley@sanaria.com, yabebe@sanaria.com, ksim@sanaria.com, slhoffman@sanaria.com, and trichie@sanaria.com. Sky Chen, StatPlus, Inc. Taipei, Taiwan, E-mail: sky.chen@statplus.com. Lenny L. Ekawati and J. Kevin Baird, Oxford University Clinical Research Unit Indonesia, Jakarta, Indonesia, and Centre for Tropical Medicine & Global Health, Nuffield Department of Medicine, University of Oxford, Oxford, United Kingdom, E-mails: lekawati@oucru.org and kbaird@oucru.org. Erni J. Nelwan, Faculty of Medicine, Universitas Indonesia, Jakarta, Indonesia, and Division of Tropical Medicine and Infectious Disease, Department of Internal Medicine, Cipto Mangunkusumo Hospital, Jakarta, Indonesia, E-mail: ejnelwan@yahoo.com.” (page 900)

